# LINE-1 hypomethylation is not a common event in preneoplastic stages of gastric carcinogenesis

**DOI:** 10.1038/s41598-017-05143-0

**Published:** 2017-07-06

**Authors:** Juozas Kupcinskas, Ruta Steponaitiene, Cosima Langner, Giedre Smailyte, Jurgita Skieceviciene, Limas Kupcinskas, Peter Malfertheiner, Alexander Link

**Affiliations:** 1 Department of Gastroenterology, Lithuanian University of Health Sciences, Kaunas, Lithuania; 2 Institute for Digestive Research, Lithuanian University of Health Sciences, Kaunas, Lithuania; 3 Department of Gastroenterology, Hepatology and Infectious Diseases, Otto-von-Guericke University Magdeburg, Magdeburg, Germany; 4 Lithuanian Cancer Registry, National Cancer Institute, Vilnius, Lithuania; 5 Centre for Demographic Research, Vytautas Magnus University, Kaunas, Lithuania

## Abstract

LINE-1 hypomethylation is widely accepted as marker for global genomic DNA hypomethylation, which is a frequent event in cancer. The aim of the study was to evaluate LINE-1 methylation status at different stages of gastric carcinogenesis and evaluate its prognostic potential in clinical settings. LINE-1 methylation was analyzed in 267 tissue samples by bisulfite pyrosequencing including primary colorectal cancer tissues (T-CRC) with corresponding adjacent colon mucosa (N-CRC), gastric cancer tissues (T-GC) with corresponding gastric mucosa (N-GC), normal gastric tissues (N), chronic non-atrophic and atrophic gastritis (CG). LINE-1 methylation level was lower in both T-GC and T-CRC when compared to paired adjacent tissues. No difference was observed for LINE-1 methylation status between patients with normal gastric mucosa, CG and N-GC. LINE-1 methylation in T-GC but not N-GC tended to correlate with age. Subgroup stratification analysis did not reveal significant differences in LINE-1 methylation status according to tumor stage, anatomical location, histological subtype, differentiation grade. We observed similar overall survival data between patients with high or low LINE-1 levels. In summary, LINE-1 hypomethylation is a characteristic feature in GC but not very common in early preneoplastic stages of gastric carcinogenesis. Prognostic role of LINE-1 hypomethylation in GC patients could not be confirmed in this cohort.

## Introduction

Gastric cancer (GC) remains a major healthcare burden across the globe and ranks as the second most common cause of cancer-related mortality^1^. The disease becomes clinically apparent mostly in advanced stages leading to the poor patients’ outcomes^2^. Gastric carcinogenesis results from the accumulation of multiple factors and characterized by a step-wise process from *Helicobacter pylori* (*H*. *pylori*) induced chronic active gastritis, to atrophic gastritis with intestinal metaplasia, dysplasia and adenocarcinoma^3^. Underlying molecular alterations that progress from gastritis to gastric cancer have been explored, but the exact mechanisms and interactions with risk factors remain unclear. Identification and description of carcinogenesis-related biological processes across all stages of gastric carcinogenesis are highly desirable for translational purposes in order to improve diagnostic and therapeutic strategies^4^.

Epigenetics is a crucial element involved in regulation of genetic stability in different malignancies^5^. DNA methylation is the most extensively studied epigenetic phenomenon in a wide range of diseases^6^. Global hypomethylation refers to decrease in DNA methylation across the entire genome and is linked with genetic instability and procarcinogenic events^7^. Hypomethylation of the entire genome partially results from demethylation in repetitive elements that account for about a half of the human genome. This is essential in gene regulation and genomic stability^8^. Long Interspersed Nucleotide Element 1 (LINE-1) is one of the major genetic elements which constitute ~17% of the genome^8^. CpG sites located within LINE-1 and their methylation levels correlate with the global genomic DNA methylation status^9^. Therefore, LINE-1 status is frequently used as surrogate marker for estimation of global DNA hypomethylation^10^. LINE-1 hypomethylation has been frequently reported in different types of cancer especially colorectal cancer (CRC)^5, 11, 12^. Furthermore, LINE-1 methylation status has been suggested as a potential biomarker for cancer detection and disease outcomes^11, 13, 14^.

LINE-1 methylation levels in tissues and blood samples of gastric cancer patients have been analyzed in several studies previously suggesting lower LINE-1 methylation as a characteristic event in GC^12, 14–17^. Of the relevance was the finding that LINE-1 hypomethylation may be associated with poor survival in Asian patients with GC^12, 18^. The analysis of LINE-1 methylation levels in DNA samples derived from blood of GC patients suggests furthermore potential diagnostic implications^16, 17^. To date, there have been several attempts to define LINE-1 methylation levels in premalignant lesions. Some of them showed a gradual hypomethylation across preneoplastic stages with gradual progression during gastric carcinogenesis^18–20^. The data on LINE-1 methylation status across different stages of gastric carcinogenesis are still limited. Most of reported studies have been conducted on Asian GC patients, while data on LINE-1 methylation levels in European subjects with GC and premalignant gastric lesions is largely unexplored^14^. It is also worth pointing out that several studies have already employed pyrosequencing method for LINE-1 analysis in GC, which is considered very robust technical modality used for LINE-1 methylation analyses^21, 22^.

The aim of the present study was to perform a comparison analysis on LINE-1 methylation level in gastric carcinogenesis. First, we compare LINE-1 methylation in tumor and non-tumor tissues in GC and CRC patients. Further, we elucidate the changes in preneoplastic conditions and compared them to methylation in normal mucosa. For the evaluation of the prognostic role of LINE-1 methylation, we performed survival analyses.

## Results

### LINE-1 methylation in CRC and GC

**Figure 1:**
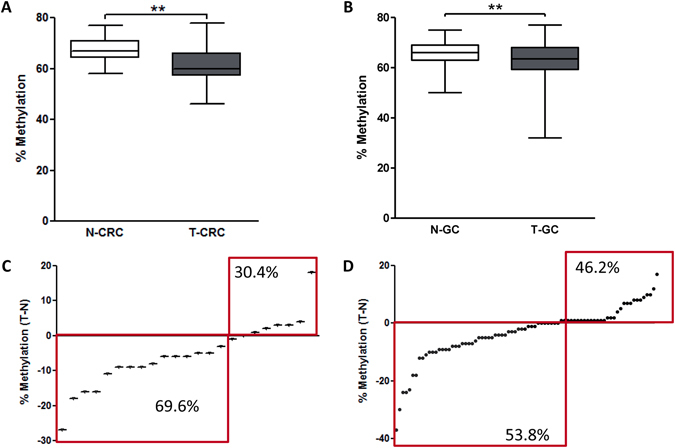
Quantitative LINE-1 methylation analyses in paired colorectal (CRC) and gastric cancer (GC) tissues. (**A**) LINE-1 methylation in paired T-CRC and adjacent N-CRC tissues (n = 24) (p = 0.0005). (**B**) LINE-1 methylation level in T-GC and adjacent N-GC tissues (n = 80) (p = 0.002). (**C** and **D**) Absolute difference between LINE-1 methylation in matching (**C**) T-CRC and N-CRC, and (**D**) T-GC and N-GC tissues. Wilcoxon test has been used for paired analyses **P < 0.005.

Methylation status in LINE-1 has been extensively studied in CRC; therefore, we included a group of patients with CRC for comparative analysis (Table 1). We performed quantitative LINE-1 methylation analysis in a cohort of paired primary CRC tissues (T-CRC) with corresponding adjacent tumor-free colonic mucosa (N-CRC). Lower LINE-1 methylation was found in T-CRC compared to N-CRC (mean ± SD: 61.15 ± 6.38% vs. 67.17 ± 4.84%, respectively, p = 0.0005; Fig. 1A). In patients with GC, LINE-1 methylation level was also lower in T-GC tissues compared to adjacent N-GC (62.48 ± 8.15% vs. 65.73 ± 4.56%, respectively, p = 0.002; Fig. 1B). Absolute number of tissues with lower LINE-1 methylation in tumorous tissue compared to non-tumorous was higher in CRC compared to GC (69.6% vs. 53.8%, respectively) (Fig. 1C and D).

**Table 1:** Characteristics of patients included in the LINE-1 methylation analysis: controls, gastritis, gastric cancer and colorectal cancer patients.

Total		Gastric cancer	Colorectal cancer	Chronic/atrophic gastritis	Controls
		n(%)	n(%)	n(%)	n(%)
		n = 80	n = 24	n = 37	n = 19
Age	mean (SD)	65.9 (11.7)	69.3 (8.8)	57.3 (13.0)	49.1 (14.9)
Gender	Female	33 (41.3)	11 (45.8)	25 (67.6)	12 (63.2)
	Male	47 (58.7)	13 (54.2)	12 (32.4)	7 (36.8)
Tumor localization	Cardia	8 (10.0)	—	—	—
	Corpus	44 (55.0)	—	—	—
	Antrum	28 (35.0)	—	—	—
	Proximal colon	—	9 (37.5)	—	—
	Distal colon	—	15 (62.5)	—	—
TNM staging	I	15 (18.7)	2 (8.3)	—	—
	II	21 (26.3)	10 (41.7)	—	—
	III	36 (45.0)	6 (25.0)	—	—
	IV	8 (10.0)	4 (16.7)	—	—
	Unknown	—	2 (8.3)	—	—
T	1/2	17 (21.3)	2 (8.3)	—	—
	3	36 (45.0)	18 (75.0)	—	—
	4	27 (33.7)	1 (4.2)	—	—
	Unknown	—	3 (12.5)		
N	0	28 (35.0)	13 (54.2)	—	—
	1	15 (18.7)	7 (29.2)	—	—
	2	13 (16.3)	2 (8.3)	—	—
	3	22 (27.5)	—	—	—
	Unknown	2 (2.5)	2 (8.3)		
M	0	72 (90.0)	8(33.3)	—	—
	1	8 (10.0)	4 (16.6)	—	—
	Unknown		12 (50.0)		
G	1 and 2	31 (38.8)	18 (75.0)	—	—
	3	49 (61.2)	3 (12.5)	—	—
	Unknown	—	3 (12.5)	—	—
Lauren’s classification	Diffuse	44 (55.0)	—	—	—
	Intestinal	25 (31.3)	—	—	—
	Mixed	7 (8.7)	—	—	—
	Unknown	4 (5.0)	—	—	—
*H*. *pylori* infection	Positive	17 (21.3)	—	25 (67.6)	—
	Negative	8 (10.0)	—	12 (32.4)	19 (100)
	Unknown	55 (68.7)	—	—	—

### LINE-1 methylation in preneoplastic gastric mucosa

**Figure 2:**
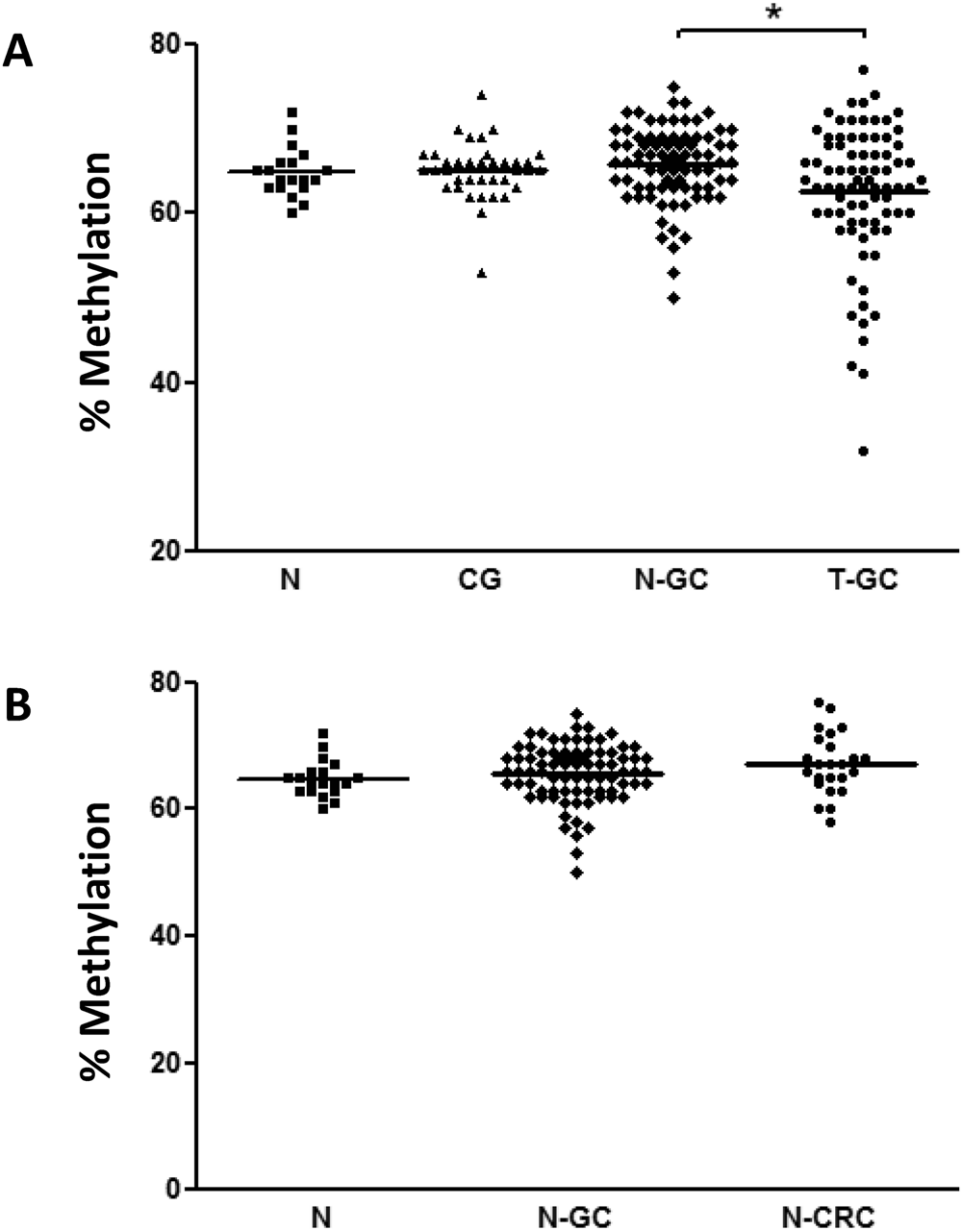
Quantitative LINE-1 methylation analyses in gastric and colon tissues. (**A**) LINE-1 methylation values were obtained using bisulfite pyrosequencing in gastric tissues from controls (N), patients with chronic gastritis (CG), adjacent non-tumor tissues (N-GC) and gastric cancer tumor tissues (T-GC) (p > 0.05). (**B**) LINE-1 methylation level comparison between normal gastric (N), adjacent non-tumor gastric tissues (N-GC) and non-tumoral adjacent colon tissue (N-CRC) (p = 0.2). Statistical analyses where performed using Mann-Whitney test *P < 0.05.

One of the major aims of our study was to evaluate LINE-1 methylation status at different stages of gastric carcinogenesis. For this reason, we performed LINE-1 methylation analysis in individuals with normal gastric mucosa without *H*. *pylori* infection and in patients with chronic atrophic gastritis. We found that methylation of LINE-1 did not differ significantly between normal tissues (N), chronic gastritis group (CG/AG) and tumor-adjacent (N-GC) gastric mucosa (mean ± SD: 64.48 ± 2.93%, 65.08 ± 3.37%, 65.75 ± 4.56% (p > 0.05), respectively) (Fig. 2A). Furthermore, we compared the LINE-1 methylation level between chronic atrophic gastritis (AG) with intestinal metaplasia and CG but no significant difference was found (data not shown), suggesting that LINE-1 methylation is rather a rare event in early stages of Correa’s cascade in gastric carcinogenesis (Fig. 2A). The only significant difference among gastric tissues with respect to LINE-1 methylation status was observed between N-GC and T-GC samples as described above (Fig. 2A). Analysis of LINE-1 methylation between N, N-GC and N-CRC revealed similar level, suggesting that LINE-1 may have relatively stable methylation pattern in GI tract in non-malignant tissues (Fig. 2B).

### LINE-1 methylation correlation analysis

**Figure 3:**
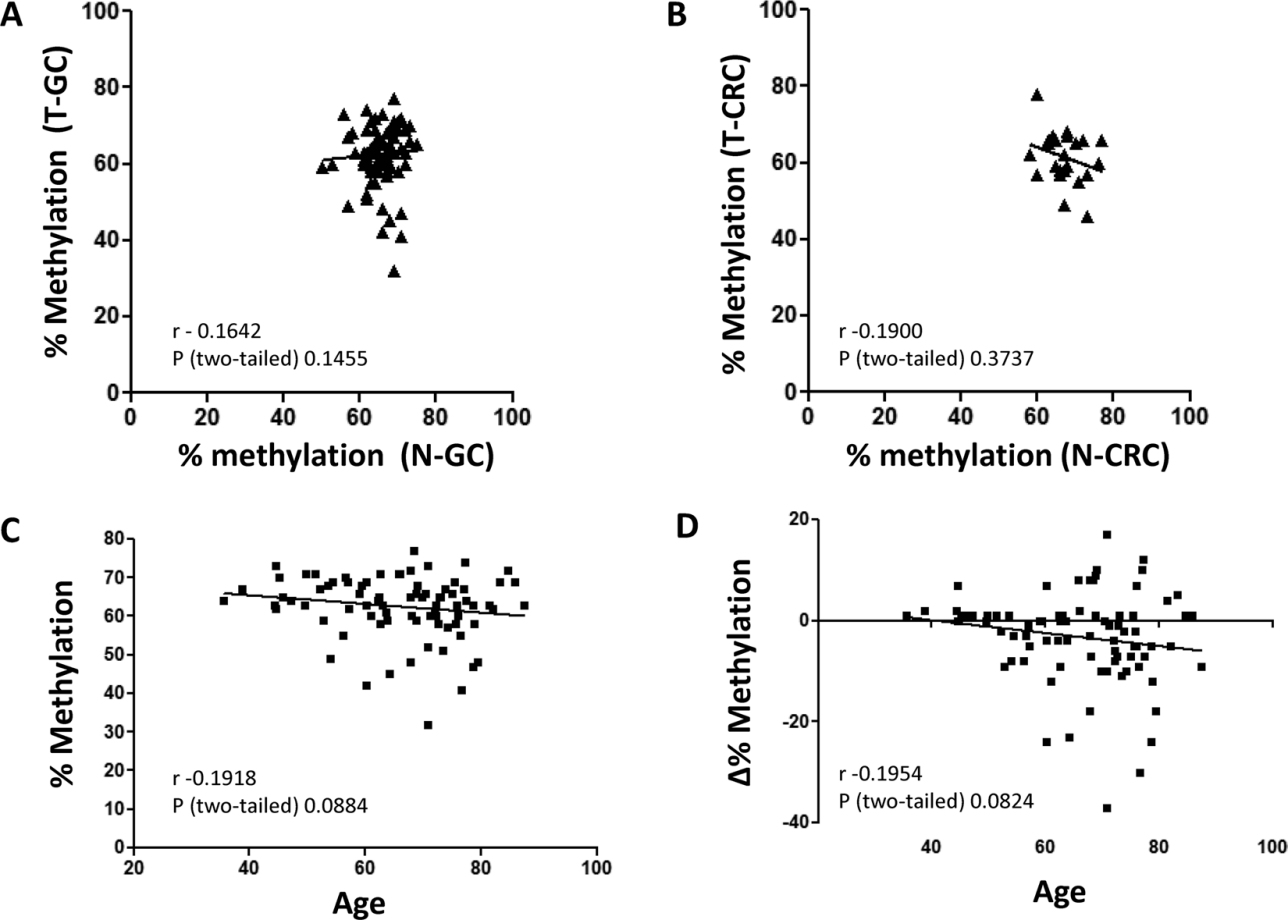
Correlation between LINE-1 methylation status in tumorous and adjacent non-tumorous tissue. LINE-1 methylation obtained using bisulfite pyrosequencing did not correlate between (**A**) N-GC and T-GC and (**B**) N-CRC and T-CRC tissues. (**C** and **D)** Correlation analysis between patient’s age and (**C**) absolute LINE-1 methylation in T-GC; and (**D**) difference in LINE-1 methylation between T-GC and N-GC tissues. Analyses were performed using Spearman’s test.

We further analyzed whether lower LINE-1 hypomethylation occurs simultaneously in tumorous and tumor-adjacent tissues. Analysis for LINE-1 methylation status in GC and CRC revealed no significant correlation between N-GC and T-GC (r = 0.16, p = 0.15, Fig. 3A) and between N-CRC and T-CRC (r = 0.19, p = 0.37, Fig. 3B), suggesting that global hypomethylation might be a focal tumor-specific event of cancerous tissues. In the next step, we evaluated the link between methylation level and patients’ age. A trend towards significant correlation was observed between patient’s age and LINE-1 methylation levels in T-GC tissue (r - 0.1918; p = 0.0884; Fig. 3C) and difference in LINE-1 methylation between T-GC and N-GC tissues (r - 0.1954; p = 0.084; Fig. 3D), however, the results did not reach the level of statistical significance.

### LINE-1 methylation in GC subgroups

**Figure 4:**
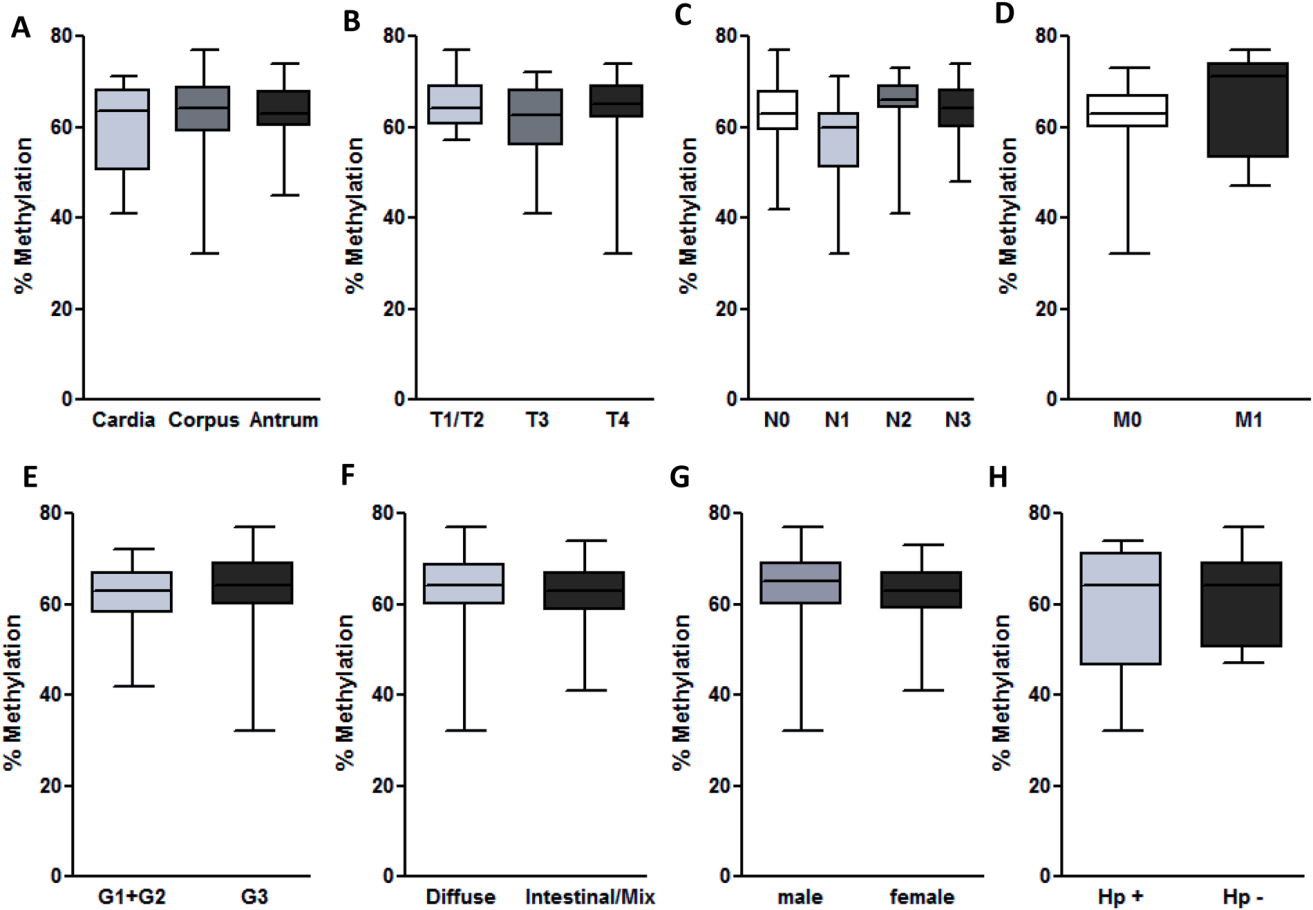
Subgroup analyses of LINE-1 methylation in gastric cancer patients according to clinicopathological data. LINE-1 methylation analyses based on (**A**) anatomical tumour localization (p = 0.41), (**B**) T- (p = 0.20), (**C**) N-, (p = 0.11) and (**D**) M-tumor staging (p = 0.17). (**E**) LINE-1 methylation differences in patients with low and high-grade tumors (p = 0.26). LINE-1 methylation differences in GC patients according to (**F**) Lauren’s classification of GC type (p = 0.39), (**G**) gender (p = 0.83) and (**H**) *H*. *pylori* status (p = 0.70). Statistical analyses were performed using Mann-Whitney for two and Kruskal-Wallis test with Dunn’s posttest for multiple comparison analyses.

LINE-1 methylation in GC samples with different clinical and pathological characteristics are presented in Fig. 4. Hypomethylation level were similar in tumors arising from different anatomical sites of stomach such as cardia, corpus and antrum (Fig. 4A, p = 0.41). No difference was found between more and less advanced stages of GC (Fig. 4B–D; T- (p = 0.20), N- (p = 0.11) and M-tumor staging (p = 0.17) or tissues with low/medium (G1/2) and poorly differentiated (G3) tumors (p = 0.26; Fig. 4E). LINE-1 methylation status was similar between histological subtypes of GC – intestinal vs. diffuse (61.84 ± 7.97% and 63.32 ± 7.78%, respectively, p = 0.39, Fig. 4F). We also found no differences with respect to gender (p = 0.83) or preexisting *H*. *pylori* infection (p = 0.70), but these sub-analyses were limited by availability of the clinical/serological data for patients with GC within the study (Fig. 4G and H).

### LINE-1 methylation and overall survival

**Figure 5:**
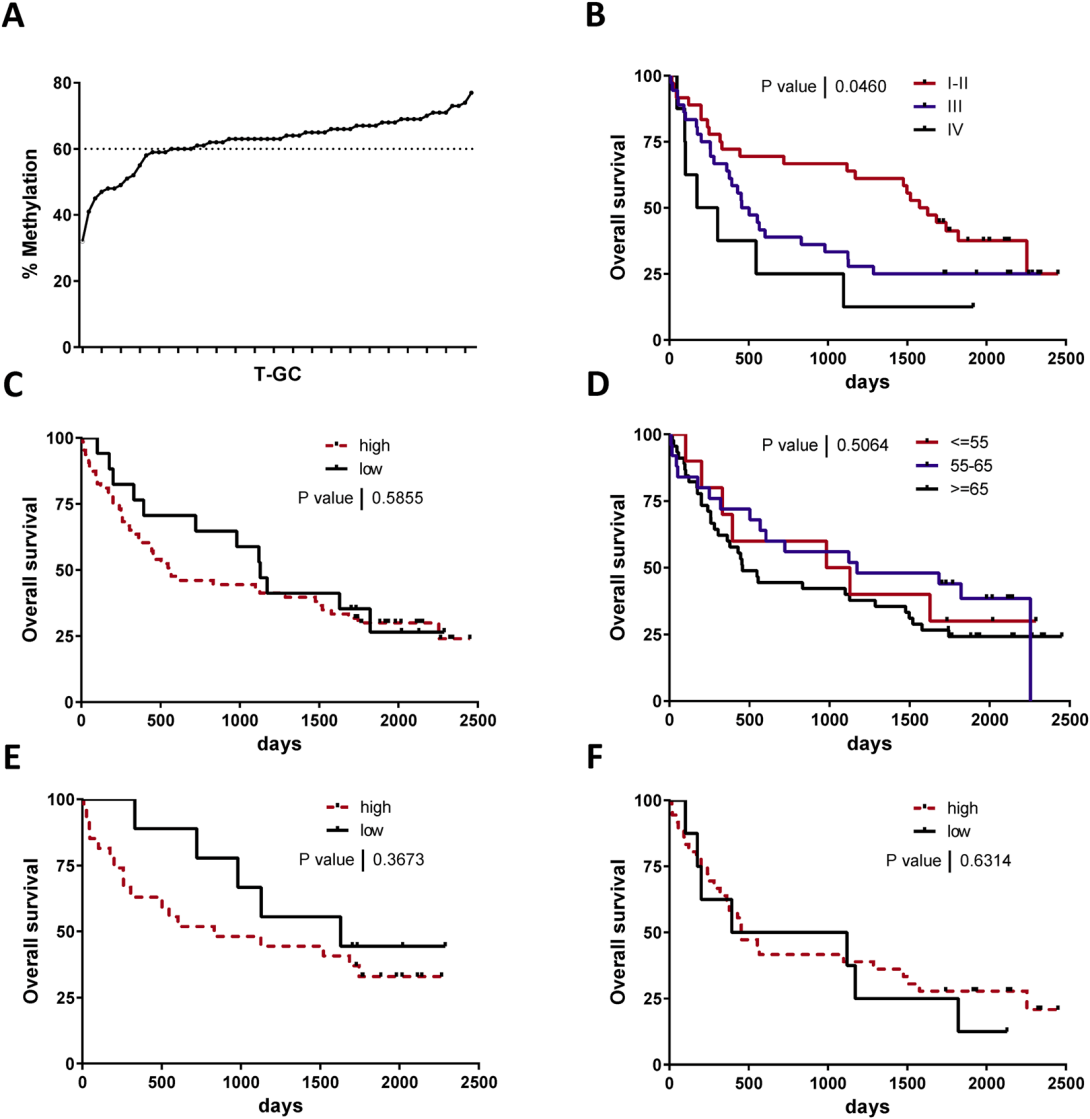
Overall survival analyses of patients with GC based on LINE-1 methylation. (**A**) Patients with GC with high and low LINE-1 methylation status defined by cut-off 60% based on LINE-1 methylation in T-GC sample (low LINE-1 methylation 21.5%). (**B**) Kaplan-Meier analyses based on UICC tumor stage with significant surivival difference among the groups. (**D**) Kaplan-Meier analyses based on high (n = 63) and low (n = 17) LINE-1 methylation status (cut-off methylation 60%) (p = 0.59). (**D**) Kaplan-Meier analyses based on high ≥ 65% (n = 45), middle > 55% and <65% (n = 25) and low ≤55% (n = 10) LINE-1 methylation status (p = 0.51). (**E**) Kaplan-Meier analyses of survival difference based on LINE-1 methylation status in intestinal/mixed type GC (p = 0.36) and in (**F**) diffuse type GC (p = 0.63). Statistical comparison between curves was performed with Log-rank (Mantel-Cox) test.

Survival data for all 80 GC patients were available for analysis. The average overall survival time after disease onset was estimated to be 1015 days (range 9–2451 days). 60% cut-off value was selected to discriminate patients with high and low LINE-1 methylation based on the previous publications and the LINE-1 methylation distribution in our set of data (21.5% of samples with low methylation)^23, 24^ (Fig. 5A). Overall, there was a significant survival difference dependent on UICC stage (Fig. 5B), confirming the validity of the survival data in our cohort. We found no differences in survival between the patients with low compared to high LINE-1 methylation (Fig. 5C, p = 0.59). This was also true if we used the more stringent cut-off of 55 creating three groups with low, middle and high LINE-1 methylation groups (Fig. 5D). Survival analyses stratified by histological GC subtype also revealed no differences in survival (Fig. 5E and F).

## Discussion

Findings of our study provide a detailed characterization of LINE-1 methylation status across preneoplastic and neoplastic stages of gastric carcinogenesis. LINE-1 hypomethylation did not differ significantly between normal gastric mucosa, chronic gastritis and tumor-adjacent tissues and was rare in preneoplastic mucosa, suggesting LINE-1 methylation predominantly as a late event in gastric carcinogenesis. More importantly, patients with low LINE-1 methylation in GC tissues showed no difference in overall survival compared to patients with high LINE-1 methylation.

Global DNA hypomethylation and CpG island promoter hypermethylation are characteristic features of various tumors^13, 25^. For instance, we have previously reported site-specific CpG island promoter hypermethylation of miR-137 in GC tissue samples, which was inversely correlated with LINE-1 methylation status^25^. In the present study, in the line with the previous reports, we confirmed decreased methylation of LINE-1 in T-CRC compared to N-CRC^26, 27^. In concordance to the results of other groups, similar observation was made for T-GC in comparison to LINE-1 methylation in N-GC (Table 2 **)**. For instance, in sporadic GC, both microsatellite stable (MSS) and unstable (MSI) GC tumors had lower LINE-1 methylation levels in tumorous tissues when compared to normal healthy mucosa^15^. Two larger studies from Japan and South Korea also showed lower LINE-1 methylation level in gastric cancer compared to matched non-tumorous gastric mucosa^12, 18^. Although the absolute LINE-1 methylation levels differed between above mentioned studies, the difference may be explained by potential confounding factors including methodological approach, sample bias or region where the study was performed^14^.

**Table 2:** Summary of the studies related to LINE-1 methylation in gastric cancer patients.

First Author	Year	Tissue origin	Tissues	Micro-dissection	Gastric cancer	Paired samples	Healthy controls	Preneoplastic/-cancerous stages	*H*. *pylori* status	Methods	LINE-1 in GC	Survival analysis	Ref.
Kupcinskas *et al*.	2017	Europe (mix)	fresh-frozen	no	80	yes	19	37	yes	PyroSeq	↓ in GC	**↔**	
Song *et al*.	2016	Korea	FFPE	yes	434	no	no	no	no	PyroSeq		**↓**	34
Kim *et al*.	2016	Korea	FFPE	yes	no	no	no	89	yes	COBRA	↓ in HGIEN		40
Kosumi *et al*.	2015	Japan	FFPE	yes	87	yes	17	20	yes	PyroSeq	↓ in GC		41
Yang *et al*.	2014	Korea	FFPE, fresh-frozen	yes	88/115	no	22	39	yes	PyroSeq	↓ in GC		42
Shigaki *et al*.	2013	Japan	FFPE	yes	203	yes	no	no	no	PyroSeq	↓ in GC	**↓**	12
Saito *et al*.	2012	Japan	fresh-frozen	no	101	yes	83	82	yes	qMSP	↓ in GC		43
Pavicic *et al*.	2012	Europe (Finland)	FFPE	yes	58	yes	no	no	no	COBRA MS-MLPA	↓ in GC		15
Bae *et al*.	2012	Korea	FFPE	yes	198/59	no	no	190	yes	PyroSeq	↓ in GC/ adenoma	**↓**	18
Balassiano *et al*.	2011	Europe (mix)	FFPE	no	98/20	yes	15	no	no	PyroSeq	↓ in GC		44
Lee *et al*.	2011	Korea	FFPE	yes	53	yes	no	79	yes	COBRA	↓ in GC/ HGIEN		20
Yoshida *et al*.	2011	Japan	fresh-frozen	no	52	no	34	76	yes	PyroSeq	↓ in GC		45
Park *et al*.	2009	Korea	FFPE	yes	59	no	no	143	yes	COBRA	↓ in GC		19

qMSP: quantitative MSP (real-time); NA: non-available; FFPE: formalin-fixed paraffin-embedded tissue; PyroSeq: pyrosequencing; GC: gastric cancer; HGIEN: high-grade intraepithelial neoplasia; Ref: references.

Contrary to previous reports, our study has revealed that LINE-1 methylation level did not differ significantly between normal gastric tissue, CG and N-GC. Using a COBRA method, Park *et al*. found lower LINE-1 methylation already in preneoplastic lesion including CG^19^. A study by Bae *et al*. reported that LINE-1 methylation decreased during the transition from intestinal metaplasia to gastric adenoma while no further decrease occurred during the transition from gastric adenoma to GC as determined by pyrosequencing technique^18^. High-grade dysplasia had significantly lower LINE-1 methylation level compared to low-grade dysplasia and this difference was associated with high diagnostic sensitivity and specificity^20^. Unfortunately, histologically confirmed adenoma or dysplasia of the stomach are quite rare in European countries, therefore we could not address this issue in our work. Because of this limitation, we cannot exclude certain degree of LINE-1 methylation changes in early neoplastic stages. Furthermore, it is also possible that with larger number of samples with preneoplastic conditions we could potentially identify smaller changes, however, the fact that LINE-1 methylation levels were similar in N, CG, AG and N-GC and the range of methylation was quite constant this rather supports our conclusions. Another factor that needed to be taken into consideration is the difference in confounding factors (exp. diet) between Asian and European cohorts. This may contribute to pronounced alterations in LINE-1 during the earlier stages of gastric carcinogenesis^28^. It is important to mention that in our cohort we had a quite a large number of diffuse-type GC cases (55%). Diffuse histological sub-type of GC may often arise from normal gastric mucosa in the absence of premalignant gastric conditions^29^ and direct comparison to molecular alterations in premalignant gastric lesions might be flawed. At this stage we can solely speculate for the difference in epigenetic “field defect” between diffuse and intestinal subtypes of GG.

In subgroup analysis, LINE-1 methylation analyses revealed no significant differences among analyzed subtypes including GC with different anatomical styles, various stages of GC, differentiation level. Furthermore, LINE-1 methylation status was similar between intestinal and diffuse subtypes of GC. Our results are supported by the study by Shigaki *et al*., where the authors also found no difference while others did show the difference^12, 18^. Because of this heterogeneity, the biological implication is probably questionable, although additional large studies may be needed to identify potential confounding factors.

Two studies in Asian population analyzed LINE-1 methylation level in regard of *H*. *pylori* infection where no association could be identified^18, 19^. Our results confirm those data, showing missing association for both tumoral and non-tumoral tissues, which is also in concordance with our results to gastritis/preneoplastic conditions. On the other hand, Yamamoto *et al*. showed significantly reduced level of LINE-1 methylation in gastric mucosa of patients with enlarged-fold gastritis, which is strongly associated with *H*. *pylori* infection^30^. Taking together, the direct impact of *H*. *pylori* infection is still not fully understood. For instances, the strong infiltration of inflammatory cells of the mucosa due *H*. *pylori* could have an impact on global LINE-1 methylation while being different in damaged preneoplastic mucosa.

In our study, we observed a trend towards negative correlation between patient’s age and LINE-1 hypomethylation in GC tissue. Bae *et al*. have reported a similar negative correlation between LINE-1 methylation level of GC and the patient age in male but not in female patients^18^. Another study assessing age-dependent hypomethylation suggested that age was negatively associated with methylation levels of Alu, but not LINE-1^31^. Since the highest risk of GC is in older population, we speculate that age-associated global hypomethylation may contribute to gastric carcinogenesis, however, this point need to be addressed in specifically designed studies.

Hypomethylation has been linked to the worse overall survival of the patients with multiple tumors including CRC, liver, lung and ovarian cancers^32^. Nevertheless, the exact mechanism is not fully understood. Opposite correlation has been, however, demonstrated in melanoma where LINE-1 hypomethylation was associated with a favorable outcome^33^. In our cohort of patients, we observed no differences in overall survival between the patients with low or high LINE-1 methylation. This was also true for different GC subtypes. Our results do not support existing data to prognostic role of LINE-1 in GC patients. For instance, LINE-1 hypomethylation was significantly associated with shorter overall survival in large cohort of GC patients from Japan^12^ and South Korea^18^. Higher proportion of patients with diffuse GC according to Lauren’s classification could be one of the explanation. Another explanation may be the difference in tumor biology between tumor in Europe and Asia. Majority of previously published papers come from Asian countries with predominantly intestinal type GC patients included in the studies ranging from 39% to 64% in study populations^12, 18^. Large number (55% of all cancer cases) of GC cases in our study were diffuse-type according to Lauren’s classification. At least applicable for our European population, our results do not support the prognostic value of LINE-1 methylation in GC patients.

LINE-1 methylation status in gastric cancer and premalignant gastric conditions among European subjects remains poorly investigated and our study provides valuable insights for perception of stepwise development of GC. Here, we performed a systematic analysis of the literature to the topic of LINE-1 methylation in gastric cancer. Table 2 summarizes the differences between various studies including tissue origin, performance of microdissection, applied methods and main output. While three studies show an association between LINE-1 methylation and worse prognosis, in our European cohort we failed to confirm those results. Although, this could be related to specific tumor biology, there is also several other factors that need to be mentioned. In comparison to survival studies from Asia^12, 18, 34^, we analyzed surgically- or endoscopically-obtained samples without prior microdissection; therefore, we could not evaluate the purity of the tumor. This limitation does not allow direct comparison to existing studies since the proportion of tumor cells (in particular in diffuse gastric cancer cells) may be variable. In similar fashion, we did not perform microdissection of epithelial cells in preneoplastic and the small amount of LINE-1 hypomethylation is still possible. Nevertheless, our results are important from the translational point of view highlighting the potential limitation of LINE-1 methylation analysis in everyday clinical praxis.

Overall, our results confirm that LINE-1 hypomethylation is characteristic feature in GC tissues. Since only marginal difference in LINE-1 hypomethylation was observed in preneoplastic tissues, we conclude that the global hypomethylation may be rather an end stage event in gastric carcinogenesis. In this European cohort of patients, LINE-1 methylation showed no association to an overall survival of GC patients.

## Materials and Methods

### Tissue samples

Tissue samples were collected at two clinical centers: Department of Gastroenterology and Surgery, Hospital of Lithuanian University of Health Sciences (Kaunas, Lithuania) and Department of Gastroenterology, Hepatology and Infectious Diseases Otto-von-Guericke University (Magdeburg, Germany) under the frame of the ERA-Net PathoGenoMics project. The study protocol was approved by Kaunas Regional Biomedical Research Ethics Committee (Protocol Nr. 8/2011) and by the Institutional Review Board of Otto-von-Guericke University Magdeburg (Protocol Nr. 80/2011). The study was performed according to the guidelines of Declaration of Helsinki. All patients participating in the study have signed an informed consent form.

### Study design

Study design and tissue collection protocol has been partly described in the previous study^25, 35^. For the LINE-1 methylation analyses, we had available 267 tissue specimens (biopsies and surgical material) including: 80 GC tumor tissues (T-GC) with corresponding adjacent non-tumorous gastric mucosa (N-GC) from GC patients; normal gastric mucosa tissue from 19 controls (N); 37 gastric antrum tissues from patients with chronic non-atrophic and atrophic gastritis with/-out intestinal metaplasia (CG); 24 primary CRC tumor tissues (T-CRC) with corresponding adjacent non-tumorous colonic mucosa (N-CRC). N and CG samples were obtained during upper GI endoscopy and were characterized histologically according to the updated Sydney classification^36^; the presence of *H*. *pylori* was additionally investigated by serology (ELISA IgG test, Virion\Serion GmbH, Germany or Helicobacter pylori IgG ELISA Kit Biohi, Helsinki, Finland) and microbiological analysis as reported previously^37^. Histological subtypes of GC patients were determined using Lauren’s criteria. All tissue samples were histologically examined and it was confirmed as non-tumorous or tumorous tissue. Biopsies from the patients N/CG/AG were obtained during endoscopy and same region samples were used for histological evaluation and methylation analysis. Tissue samples for methylation studies were immediately snap-frozed in liquid nitrogen and stored at −80 °C until analyses. Detailed characteristics of the subjects included in the study are presented in Table 1.

### Survival analyses

The survival data of 80 GC patients were retrieved from Lithuanian Cancer Registry and medical records at Hospital of Lithuanian University of Health Sciences. The time interval between the date of GC onset and the date of death was defined as overall survival of GC patients. The patients, who were still alive at the moment of data collection, were censored as dead as for 28th February, 2017. For survival analysis we used a cut-off value of 60% at LINE-1 (high *vs*. low methylation levels). This selection was based on the observation of several previously published papers where cut-off of 55/65% has been suggested as appropriate to define the subjects with global hypomethylation^23, 24^. Survival data were analyzed using Kaplan-Meier survival curves.

### DNA isolation

DNA for methylation analyses from tissue samples was extracted as described previously^25^. Briefly, DNA was isolated with QIAzol Lysis reagent and chloroform, using the interphase, according to user-developed protocol (QIAGEN, Valencia, CA, USA). Qualitative and quantitative testing of extracted DNA samples was performed spectrophotometrically using Biophotometer (Eppendorf, Germany).

### DNA methylation analyses

Bisulfite conversion of purified genomic DNA was performed using Cells-to-CpG™ Bisulfite Conversion Kit (Life Technologies) according to the manufacturer’s protocol. After PCR using biotin-labeled LINE-1 region primers, the success of reaction was verified in agarose gel (1%) electrophoresis and no-template controls. For quantitative methylation analyses we used bisulfite pyrosequencing of LINE-1, which was performed on PyroMark Q96 ID (QIAGEN) using PyroMark® Gold Q96 reagents (QIAGEN) according to manufacturer’s instructions. As previously described, we accessed LINE-1 X58075 103–249 bp region with mean of 4 CpG-sites^38, 39^. LINE-1 primers: forward TTTTGAGTTAGGTGTGGGATATA, reverse 5′-biotin-AAAATCAAAAAATTCCCTTTC and pyrosequencing AGTTAGGTGTGGGATATAGT. Briefly, biotin-labeled PCR products were first captured on streptavidin-coated magnetic beads and then underwent pyrosequencing procedure. Mean methylation level of 4 measured CpG sites was used for the further analyses. Samples with poor DNA quality and/or repeatedly insufficient bisulfite conversion were excluded from further analyses.

### Statistical analysis

The statistical analyses were performed using GraphPad Prism 6.0 statistical software (San Diego, CA, USA). Data were presented as mean % methylation ± standard deviation (mean ± SD) and absolute numbers with proportions (n, %) where appropriate. Quantitative variables for nonparametric analyses were performed using Wilcoxon text for paired and Mann-Whitney U test for unpaired analyses. For multivariate analyses, we used Kruskal-Wallis test with Dunn’s multiple comparison *post test*. Correlation analyses were performed using Spearman’s Test, and Log-rank (Mantel-Cox) test was used to compare survival curves. Two-sided p-values of <0.05 were considered statistically significant in all tests.
